# Chicago Medical Society

**Published:** 1889-04

**Authors:** 


*Stated Meeting, March 18, 1889.*

THE PRESIDENT IN THE CHAIR.

PROFESSOR HENRY M. LYMAN made some remarks on the

ETIOLOGY OF NEURALGIA, which appear in another part of THE JOURNAL AND EXAMINER.

PROFESSOR DANIEL R. BROWER read a paper on

THE DIFFERENTIAL DIAGNOSIS OF NEURALGIA,

(See this issue of THE JOURNAL.)

DR. H. N. MOYER read a paper on the

TREATMENT OF NEURALGIA, in which he said it was the result of four or five years experience with the use of certain drugs. Remedies must be directed to the ætiology and conditions which underlie the system. Morphia, by reason of its powerful anodyne effects, must rank foremost in the treatment of neuralgia. Antipyrin must be placed second because of its powerful analgesic effect. A simple, but by no means to be despised remedy, is the injection of cold water under the skin. He had found this method more uniformly successful than was generally supposed. The injection should be made as nearly as possible over the point of exit of the effect nerve or bony or fibrous canal. If asked what remedy had been most useful in the treatment of chronic and inveterate cases of neuralgia, he would say aconitine. Next to aconitine was quinine, which was a very valuable remedy in those cases which manifest decided periodicity, and in neuralgias of a distinctly malarial origin, it is the remedy *par excellence.* Hypodermic injections of strychnia are useful. Arsenic is a remedy greatly extolled, its greatest usefulness being in the malarial forms of the trouble, and it is necessary to resort to it sometimes in those cases in which quinine fails. Thein might be mentioned, and unlike some of the before mentioned drugs, it seems to exert its principal action upon the peripheral nervous system, and theoretically it should be useful in the peripheral forms of neuralgia. Electricity should not be lost sight of in the treatment, and perhaps the form of current that is most useful is the galvanic. The polarity, however, seemed to make but little difference. The induced current may be useful in some cases, and is indicated. Static electricity produces results similar to those of the faradic current, and in some cases a cure is known to have been effected by extracting sparks from the region of the affected nerve when the patient is upon an insulated stool. Osmic acid, which has received extended notice of late, used in 30-minim doses of a one per cent. solution, injected over the site of the affected nerve, is one of the best means at the disposal of the physician. The solution should be freshly prepared, as decomposition takes place in a few hours, and then there is formation of abscess.

DR. F. C. HOTZ, in opening the discussion, said oculists had for many years been familiar with the fact that eye strain is very often associated with neuralgia of the supra-orbital and other nerves at more distant places from the eye, but as patients who consulted oculists did so mostly on account of disturbances which they directly referred to the eye, they (the oculists) had for a long time no opportunity to settle the question of the causative relation between eye strain and neuralgia. Within the past year or so, however, he thought the fact had been definitely settled that the disturbance in the functions of the eye, whether from focalizing apparatuses, their accommodation, or in the muscular adjustment of the two eyes in vision, a disturbance of the harmonious function of these apparatuses was a most prolific source of neuralgia, and it is not only the marked deviations from the normal standard of refractive and accommodative function and manifest disturbance of the harmonious action of the erectile muscles which produce headaches, but a very slight degree of this disturbance—so slight, indeed, that in many cases patients are not aware of any error of refraction or accommodation until a searching examination is made by an expert. This had been mentioned within the last five years so frequently, and had been proven in so many instances, that he thought in any discussion on the etiology of neuralgia it ought to be spoken of, because he considered this as one of the most important advancements that had been made in practical medicine. The correction of such disturbances of the eye was in many instances the remedy for the relief of the neuralgia.

Dr. Lyman Ware said that in cases of cold affecting the membrane of the ear, which does not lead to any deep inflammation, but simply catarrhal difficulties, neuralgia occurs frequently, and invariably extends to different portions of the head. He would like to emphasize what Dr. Moyer has said in the treatment regarding osmic acid. A few years ago a gentleman had persistent neuralgia located in one of his fingers. The affected member had been treated several times, and he believed a portion of the nerve had been stretched or excised without permanently relieving the neuralgia. The neuralgia returned a few months after the operation. He resorted to osmic acid, and the first injection caused the neuralgia to completely disappear. This was the only case in which he had tried it.

Dr. C. P. Pruyn said he often found the common source of neuralgia to be a calcification in the pulp of a tooth proper. There was nothing to lead one to think that it was a particular tooth that caused the neuralgia, hence the diagnosis was oftentimes made by exclusion of every known cause, and then perhaps the dentist would drill into a tooth that had every appearance of being perfectly sound and find within the bony walls this calcific deposit.

Dr. W. F. Coleman said that within the last two or three weeks he had met with three cases of neuralgia of the ear, due to reflex irritation, or that occurred through dental caries. These cases were referred to a dentist, who, after removing the caries, relieved the neuralgia in the ear.

Professor A. E. Hoadley's experience had not been very extensive in operating upon the nervous system for the relief of neuralgia. One of the greatest difficulties standing in the way of surgeons is in locating the lesion that causes the neuralgic pain. The most common, persistent neuralgia requiring operative interference, and which gives the most trouble in making a diagnosis, is probably that of the fifth nerve. He then cited two cases to illustrate the difficulties of diagnosis.

Dr. R. H. Babcock said there was a class of neuralgias that interested him, and more particularly angina pectoris, which is popularly supposed to be almost exclusively associated with organic disease of the heart. There were also cases of neuralgia of the heart which are inorganic, that is, not associated with organic disease of the organ, and these came under two heads, viz., *angina reflectoria*, and *angina vasomotoria*. He was satisfied that these two conditions were frequently associated with one or the same cause. He recalled to mind the case of a lady, 35 years of age, who is subject to attacks of angina with pronounced vaso-motor spasm, as shown by coldness of the extremities, paleness of the face, marked arterial tension, etc. This case he thinks is probably due to irritation in the pelvis. A careful examination failed to elicit any cardiac disease. Cases of inorganic angina pectoris are more likely to be found in the female than in the male sex, since the former are of a more highly nervous organization, and are subject to so many pelvic diseases which exert a specially marked influence upon the general system.

PROFESSOR FRANK BILLINGS had a theory of neuralgia, based, however, on little broader grounds, and he hoped to prove part of it in the not very distant future. He believed that more general neuralgias depended upon auto-infection in an individual than upon any other one cause, and this self-infection comes, as a rule, from the digestive tract; that it is located in the tract of a certain nerve. For instance, if there is an error of refraction, the patient will have headache; if there is ovarian difficulty the patient suffers from neuralgia connected with that part of the body. The trouble connected with the digestive tract is the cause of more neuralgias about the heart than is recognized by those who make a specialty of cardiac diseases. With inorganic heart disease there is always more or less venous congestion of the liver, stomach, spleen, and other organs connected with the portal system. If Dr. Babcock had carefully inquired into the cause of the neuralgia in the case he reported, he would find it to be associated with indigestion, brought about by a too frequent indulgence in sweets and starchy foods, thus setting up fermentation within the digestive tract.

